# Flame Retardancy of Low-Viscosity Epoxy Resins and Their Carbon Fibre Reinforced Composites via a Combined Solid and Gas Phase Mechanism

**DOI:** 10.3390/polym10101081

**Published:** 2018-09-29

**Authors:** Ákos Pomázi, Beáta Szolnoki, Andrea Toldy

**Affiliations:** 1 Department of Polymer Engineering, Faculty of Mechanical Engineering, Budapest University of Technology and Economics, 1111 Budapest, Hungary; pomazia@pt.bme.hu; 2 Department of Organic Chemistry and Technology, Faculty of Chemical Technology and Biotechnology, Budapest University of Technology and Economics, 1111 Budapest, Hungary; bszolnoki@mail.bme.hu

**Keywords:** phosphorus-containing additive flame retardants, combined solid and gas phase mechanism, low viscosity epoxy resins, carbon fibre reinforced composites

## Abstract

Low viscosity, potentially renewable aliphatic epoxy resins, appropriate for processing with injection techniques were flame retarded with the use of resorcinol bis(diphenyl phosphate) (RDP), acting predominantly in the gas phase, ammonium polyphosphate (APP), acting in the solid phase, and their combination. Samples of gradually increasing phosphorus (P) content (1%, 2%, 3%, 4%, and 5%) and mixed formulations with 2% P from APP and 2% P from RDP were prepared. The fire retardancy of matrix and carbon fibre reinforced samples was examined by limiting oxygen index (LOI), UL-94 tests, and mass loss calorimetry. The thermal stability of the matrices was investigated by thermogravimetric analysis, whereas the effect of flame retardants (FRs) on the crosslinking process and glass transition temperature was evaluated by differential scanning calorimetry in matrices and by dynamic mechanical analysis in composites. According to the results, although the trifunctional glycerol -based (GER) and the tetrafunctional pentaerythritol-based (PER) epoxy resins have a similar initial LOI and horizontal burning rate, GER has an approximately 1.5 times higher peak of heat release rate (pHRR) than PER. At least 4% P content is necessary to reach a reasonable improvement in fire performance in these resin transfer molding (RTM)-compatible systems and with the same FR-content PER reaches better fire performance. RDP has an early gas phase effect at the beginning of degradation, while later on the solid phase action of APP prevails, although in composites hindered by the reinforcing carbon fibres. In PER composites, the combination of APP and RDP had a synergistic effect, leading to a pHRR of 218 kW/m^2^ and total heat release of 18.2 MJ/m^2^.

## 1. Introduction

Carbon fibre reinforced polymer composites, which are capable of replacing metallic structures, are emerging in several high-tech sectors due to their excellent mechanical properties. However, in order to meet the strict safety requirements of demanding sectors like the aircraft and automotive industries, their main disadvantage, the flammability of the organic polymer matrix, has to be addressed [1,2]. The main challenge in improving their flame retardant (FR) properties is to simultaneously maintain their other important characteristics, such as glass transition temperature and mechanical properties, as flame retardants usually have a plasticizing effect [3,4]. Furthermore, the fire retardancy of polymers in the presence of carbon fibre reinforcement also raises a number of other concerns: the ignition of these composites is facilitated by the high thermal conductivity of the carbon fibres (this phenomenon is addressed as the candlewick effect) [5], and the applied flame retardants usually increase the viscosity of the polymer matrix, which is a key property during the production of composites by injection technologies such as resin transfer molding (RTM) commonly used in high-tech industries [6]. Furthermore, the reinforcement can filter out the solid phase flame retardants during the injection of the matrix, which may lead to non-uniform particle distribution [7,8], and consequently to uneven fire performance. And last but not least, when flame retardants acting in the solid phase are applied, it has to be taken into account that the incorporated carbon fibres interfere in their mode of action and hinder intumescent behavior [9,10,11], leading to decreased fire performance.

We investigated the fire retardancy of a trifunctional glycerol (GER) and a tetrafunctional pentaerythritol-based (PER) epoxy resin (EP) and their carbon fibre reinforced composites. Our choice of EP components was driven by two important reasons: First, these aliphatic EPs have low enough viscosity to be processed by injection molding techniques even at high FR loadings, which is important in terms of up-scaling and automatization of composite production. Secondly, although the EP components selected are presently manufactured on a mineral oil base, they can be possibly produced from renewable sources: glycerol is accessible in large quantities from natural fatty acids, while pentaerythritol can be also synthesized from bio-based methanol. As FRs, we applied ammonium polyphosphate (APP), acting in the solid phase [12], resorcinol bis(diphenyl phosphate) (RDP), acting predominantly in the gas phase [13], and their combination, which proved to be synergistic in terms of fire retardancy in previous studies of the authors [14,15]. The fire retardancy of matrix and carbon fibre reinforced samples was examined by limiting oxygen index (LOI), UL-94 tests, and mass loss calorimetry. The thermal stability of the matrices was investigated by thermogravimetric analysis, whereas the effect of FRs on the crosslinking process and glass transition temperature was evaluated by differential scanning calorimetry in matrices and by dynamic mechanical analysis in composites.

## 2. Methods, Present Situation

### 2.1. Materials Used

As EP components, we used two aliphatic components, trifunctional glycerol-based GER (MR3012, IPOX Chemicals Ltd., Budapest, Hungary; main component: triglycidyl ether of glycerol, viscosity 0.16–0.2 Pa·s at 25 °C, density 1.22 g/cm^3^ at 25 °C, epoxy equivalent 140–150 g/eq), and tetrafunctional pentaerythritol-based PER (MR3016, IPOX Chemicals Ltd., Budapest, Hungary; main component: tetraglycidyl ether of pentaerythritol, viscosity 0.9–1.2 Pa·s at 25 °C, density 1.24 g/cm^3^ at 25 °C, epoxy equivalent 156–170 g/eq). A cycloaliphatic amine MH 3122 was used as a hardener (supplier: IPOX Chemicals Ltd., Budapest, Hungary; main component: 3,3′-dimethyl-4,4′-diaminodicyclohexylmethane, amine hydrogen equivalent 60 g/eq, viscosity at 25 °C 80–120 mPas, density at 25 °C 0.944 g/cm^3^).

As flame retardants, we applied ammonium polyphosphate (APP) (supplier: Nordmann Rassmann (Hamburg, Germany), trade name: NORD-MIN JLS APP, P content: 31–32%, average particle size: 15 μm) and resorcinol bis(diphenyl phosphate) (RDP) (supplier: ICL Industrial Products (Beer Sheva, Israel), trade name: Fyrolflex RDP, P content: 10.7%).

The reinforcement was PX35FBUD030 unidirectional carbon fibre (CF) fabric consisting of Panex 35 50 k rovings, with an areal weight of 300 g/m^2^ (Zoltek Zrt., Nyergesújfalu, Hungary).

**Figure 1:**
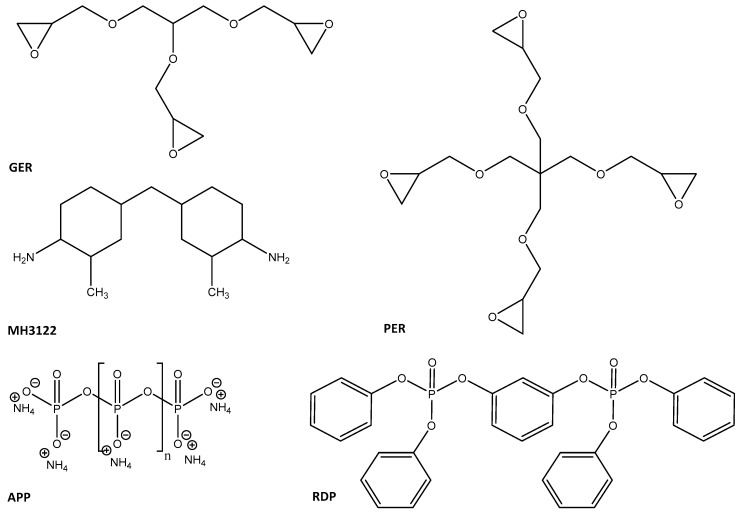
Chemical structures of the main components of the components applied: triglycidyl ether of glycerol (GER), tetraglycidyl ether of pentaerythritol (PER), 3,3′-dimethyl-4,4′-diaminodicyclohexylmethane (MH 3122), ammonium polyphosphate (APP) and resorcinol bis(diphenyl phosphate) (RDP).

The chemical structures of the EP monomers and FR additives used can be seen in Figure 1.

### 2.2. Methods

#### 2.2.1. Matrix Sample Preparation

During the preparation of the specimens, the mass ratio of the EP component and hardener was 100:40 both in the case of GER and PER. GER and PER-based EP samples of gradually increasing P content (1%, 2%, 3%, 4%, and 5%) from APP or from RDP inclusion were prepared. In addition to these samples containing only one flame retardant (FR), mixed formulations with 2% P content from APP and 2% P content from RDP were also prepared. The P content of the samples in mass% was related to the total mass of the matrix (epoxy resin + hardener + flame retardant). First, the FRs (APP, RDP, or both) were added to the EP component. Then the hardener was added and the components were mixed in a crystallizing dish at room temperature until the mixture became homogenous. The specimens were crosslinked in appropriately-sized silicon molds. The curing cycle, determined on the basis of differential scanning calorimetry (DSC), involved two isothermal heat steps: 1 h at 80 °C, followed by 1 h at 100 °C.

#### 2.2.2. Composite Sample Preparation

Epoxy resin composites with 4% P content related to the matrix were prepared. The composite laminates were made by hand lamination in a press mold. Each carbon weave layer was separately impregnated. We compressed the prepared laminates with 180 bar of hydraulic pressure (which equals to approx. 25 bar pressure on the laminate) in a T30 type platen press (Metal Fluid Engineering s.r.l., Verdello Zingonia, Italy) to achieve high and uniform fibre content in the composites. 2 mm thick laminates were made in [0]_5_ layup (5 unidirectional layers). The heat treatment was the same as in the case of the matrices, and it was carried out during pressing. The fibre content of the composites was 60 ± 1 mass%.

#### 2.2.3. Rheology

The temperature dependence of the viscosity of the resin was determined by parallel plate rheometry with a TA Instruments AR2000 device (New Castle, DE, USA) in the range of 25–80 °C, at 5 °C/min temperature ramp, and at a shear rate of 0.1/s.

#### 2.2.4. Differential Scanning Calorimetry (DSC)

The DSC tests were performed with a TA Instruments Q2000 device (New Castle, DE, USA) in 50 mL/min nitrogen flow using Tzero-type aluminum pans. The sample mass was 5–10 mg. The curing process of the samples was investigated with a three-step temperature program consisting of heat/cool/heat cycles: after a linear ramp from 25–250 °C with a heat rate of 3 °C/min (first cycle), the sample was cooled down to 0 °C at a cooling rate of 50 °C/min, followed by a second linear heating ramp from 0–250 °C at a heating rate of 10 °C/min (second cycle) so that proper conversion was achieved. The glass transition temperature (*T*_g_) values were determined from the second heating scan and were defined as the inflection point of the transition curve.

#### 2.2.5. Thermogravimetric Analysis (TGA)

The thermal stability of the samples was investigated with a TA Instruments TA Q500 device (New Castle, DE, USA) (in the range of 25–800 °C, with a heating rate of 20 °C/min, under a nitrogen gas flow rate of 30 mL/min). A platinum-HT type sample pan was used; the mass of the sample was 5–10 mg in each case.

#### 2.2.6. Characterization of Fire Behavior

The fire behavior of the samples was characterized with limiting oxygen index tests (LOI, according to ASTM D2863 (American Society for Testing and Materials (West Conshohocken, PA, USA)). The LOI expresses the lowest volume fraction of oxygen in a mixture of oxygen and nitrogen that supports flaming combustion of a material under specified test conditions. The size of the samples was 120 mm × 15 mm × 2 mm.

We also carried out standard UL-94 flammability tests (according to ASTM D3081 and ASTM D635) in order to classify the samples based on their flammability in horizontal and vertical test setups. The size of the samples was 120 mm × 15 mm × 2 mm. The increasing values of UL-94 ratings are as follows: HB, V-2, V-1, V-0.

Mass loss type cone calorimetry (MLC) tests were performed with an instrument made by FTT Inc. (East Grinstead, UK) according to the ISO 13927 standard method. 100 mm × 100 mm × 2 mm specimens were exposed to a constant heat flux of 50 kW/m^2^ and ignited. Heat release values and mass reduction were recorded during burning.

#### 2.2.7. Dynamic Mechanical Analysis (DMA)

We performed DMA tests in three point bending setup with a TA Q800 device of TA Instruments (New Castle, DE, USA) to investigate the dynamic mechanical properties and determine the glass transition temperature (*T*_g_) of the composites. The temperature range was 25–200 °C and the heating rate was 3 °C/min, the applied frequency was 1 Hz. The amplitude was strain-controlled with 0.1% relative strain. The specimen size was 55 mm × 10 mm × 2 mm (length × width × thickness), and the support span was 50 mm. The glass transition temperature obtained from the tan delta peaks (*T*_g_) and the storage modulus (*E*’) values at 25 °C and 75 °C were determined with the software of the instrument (TA Instruments Universal Analysis 2000 4.7A version).

## 3. Results

### 3.1. Preliminary Screening of the Flame Retarded Compositions Based on LOI and UL-94 Results

EP samples of gradually increasing phosphorus content (1%, 2%, 3%, 4%, 5%) were prepared for the preliminary screening of fire performance. In addition to these samples containing only one FR, mixed formulations with 2% P content from APP and 2% P content from RDP were also tested as seen in Table 1.

According to the results, although the glycerol-based and the pentaerythritol-based reference epoxy resin matrices have similar LOI (22 vs. 23 *V*/*V*%) and horizontal burning rates (27 vs. 32 mm/min, both having a HB UL-94 rate), it is evident that with the same FR-content, PER reaches better fire performance. By gradually increasing the P content in both matrices the LOI shows an increasing tendency, as expected, but to a different extent: in GER the 2% P content introduced by APP leads to an LOI of 23 *V*/*V*%, while in PER to an LOI of 32 *V*/*V*%. In the case of RDP, the differences are less pronounced, but still the overall performance of the PER samples is better. Concerning the UL-94 results, with the use of APP, all GER samples remained HB, while in PER 4% P content led to a V-1 rate, while 5% P content was enough for a V-0 rate. The application of RDP led to better UL-94 rates: in GER 4% P content was sufficient for a V-1 rate, while 5% P content led to a V-0 rate. In PER, 4% P content was enough to reach the self-extinguishing V-0 rate. As for the samples containing both APP and RDP, with a total of 4% P content, in the case of the GER matrix, the LOI was between the LOI of the GER 4% P APP and GER 4% P RDP sample, while in the case of PER, the LOI decreased by 1 *V*/*V*% compared to the PER samples containing only one FR. As for the UL-94 rates of the mixed FR samples, in both matrices the V-0 rate was achieved with 4% P content, which suggests a synergistic effect, as in GER the 4% P APP sample was HB, while the 4% P RDP was V-1, and in PER the 4% P APP sample was V-1, and only the 4% P RDP sample reached V-0 with 4% P content.

According to these results, at least 4% P content is necessary for a reasonable improvement in FR performance. Since the samples with 5% P content suffered from the softening effect due to the large amount of FR incorporated, we analyzed the compositions with 4% P content in detail.

### 3.2. Effect of the Additives on the Viscosity of Epoxy Resins Determined by Parallel Plate Rheology

Processing by injection molding requires the viscosity of the epoxy resin systems to remain preferably in the region of 100–300 mPa·s [16] even at high FR loadings.

The reference epoxy resin samples and the samples with 4% P content were subjected to parallel plate rheology in order to quantify the effect of the additive FRs on their viscosity as a function of temperature, as seen in Table 2.

The results show that GER 4% P APP and GER 2% P APP 2% P RDP samples remain injectable even at room temperature, and except the PER 4% P APP composition, which needs to be heated above 60 °C, all other systems are injectable at a temperature as low as 40 °C, which is of crucial importance concerning upscaling and automating composite production.

### 3.3. Effect of the Additives on the Crosslinking and Glass Transition Temperature of the Epoxy Resins Determined by DSC

We performed DSC analysis on the reference epoxy resin samples and the samples with 4% P content in order to quantify the effect of the additive FRs on the glass transition temperature, reaction enthalpy, and temperature of the exothermic peak in GER and PER matrix samples as seen in Table 3.

The plasticizing effect is more pronounced in the case of liquid RDP; when 4% P was added, *T*_g_ decreased by 53 °C in GER and by 33 °C in PER. In the case of solid APP, in GER the decrease was 16 °C, while in PER *T*_g_ remained unchanged. In the combined FR samples, the APP slightly compensated for the softening effect of RDP. As for the effect on the crosslinking process, the temperatures belonging to the exothermic peak of curing showed no significant differences, although the reaction enthalpy of crosslinking decreased in all cases. Yet again, due to the high ratio of RDP needed to reach 4% P content, RDP considerably reduced the reaction enthalpy both in GER and PER, as anticipated. With the intention of having a clear comparison of the effect of APP and RDP on the crosslinking process, reaction enthalpies related to the mass of epoxy resin matrix (disregarding the mass of the added FR(s)) were also compared. The results show that the effects of APP and RDP are similar, and in mixed FR samples the decrease was slightly smaller than in the systems containing only one FR.

### 3.4. Effect of the Additives on the Thermal Stability of the Epoxy Resins, Determined by TGA

The reference epoxy resin samples and the samples with 4% P content were subjected to TGA analysis and the effect of the additive FRs on thermal stability was quantified as shown in Table 4.

According to the TGA results, RDP, acting mainly in the gas phase at the beginning of degradation, decreased the temperature belonging to 5% and 50% mass loss more significantly than APP, acting in the solid phase. The results of the mixed FR samples were between the temperatures measured in the case of the systems containing only one FR. The initial maximum mass loss rate of GER is much higher than that of PER, which underlines the preliminary LOI and UL-94 results of these matrices. Except the GER 4% P APP samples, all FR systems have a reduced maximum mass loss rate of around 1%/°C. As for the char yield at 800 °C, the FRs increase the amount of solid residues in the following order: 4% P APP < 4% P RDP < 2% P APP 2% P RDP.

### 3.5. Fire Performance

#### 3.5.1. MLC Results of the Reference and Flame Retarded Epoxy Resin Matrices Containing 4% P

**Figure 2:**
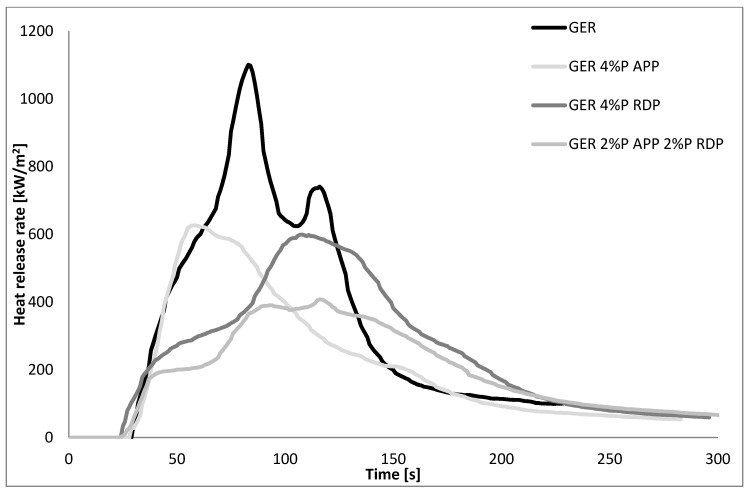
The heat release rate of the reference and flame retarded GER matrices.

**Figure 3:**
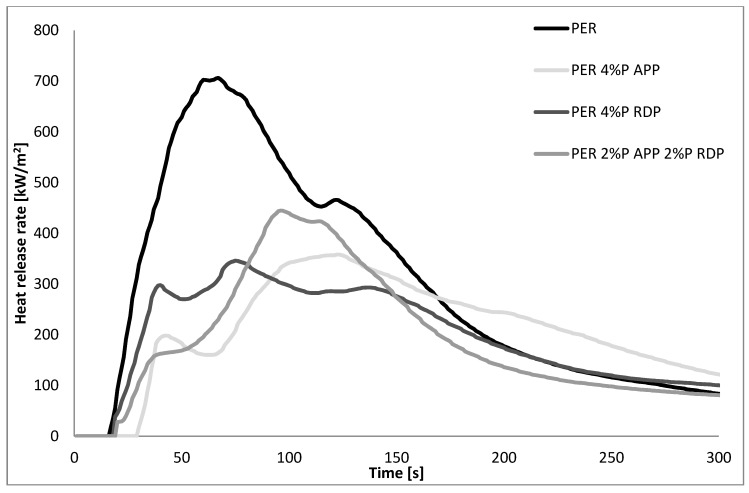
The heat release rate of the reference and flame retarded PER matrices.

The preliminary LOI and UL-94 screening of the flame retarded GER and PER matrices (see Section 3.1) show that at least 4% P content was necessary to achieve reasonable improvement, as seen in Figure 2 and Figure 3, and Table 5, therefore only compositions with 4% P content were subjected to MLC.

First of all, the comparison of the two epoxy matrices shows that the trifunctional GER has approx. 1.5 times higher pHRR than the tetrafunctional PER (1101 vs. 706 kW/m^2^), which is in good agreement with the LOI and UL-94 results—it is more challenging to reach decent FR levels in GER than in PER. The HRR curve of GER has a sharper first peak, with a smaller second peak, while in the case of PER, the first peak is wider and the second peak is rather just a shoulder on the first peak. As for the FR samples, in GER both APP and RDP significantly reduce the pHRR (from 1101 to 627 and 611 kW/m^2^, respectively). The curve of the system containing APP follows the curve of the reference up to 60 s, as this amount of APP was not enough to shift the HRR peak in time as well, just to reduce the height of the curve. In the case of RDP, acting mainly in the gas phase, not only the pHRR was reduced, but also the HRR curve was shifted in time (the time of the pHRR increased from 83 to 108 s). The comparison of the curves of the systems containing only one FR with those of the mixed FR sample shows a clear synergism: the pHRR was further reduced to 408 kW/m^2^ and shifted in time to 116 s. Compared to the reference, the mixed FR sample has a longer and lower plateau after ignition, and a longer and wider first peak with a small shoulder at the same time as the second peak of the reference appears. As for PER samples, 4% P from APP was enough not only to increase the TTI by 10 s (from 13 to 23 s) compared to the reference EP, but also to significantly reduce the height of the first peak (from 700 to 200 kW/m^2^), which was followed by a second, wider peak with a maximum of 358 kW/m^2^. Contrary to GER, in PER the 4% P from RDP was not enough to significantly delay the fully developed fire, although it reduced the HRR peaks to 298 and 346 kW/m^2^. In the case of the mixed FR PER sample, a similar plateau appeared as in the case of PER at around 165 kW/m^2^, which was followed by an elongated peak, with a slightly higher maximum than that of the PER samples containing only one FR. Nevertheless, both in PER and GER the mixed FR systems had the lowest THR value (even with the lowest amount of residue among FR samples).

#### 3.5.2. The LOI and UL-94 Results of the Reference and Flame Retarded Epoxy Resin Composites Containing 4% P

As the preliminary LOI and UL-94 screening showed, at least 4% P content was necessary for reasonable improvement in GER and PER matrices (see Section 3.1); therefore only composites with 4% P content were prepared for further fire tests.

The LOI and UL-94 results in Table 6 show that the inclusion of carbon fibres itself increased the LOI of GER and PER from 22 to 25 *V*/*V*% and from 23 to 31 *V*/*V*%, respectively. As for the effect of flame retardants, in both matrices APP led to the best results, a LOI of 31 *V*/*V*% and a HB UL-94 rate in GER and a LOI of 37 *V*/*V*% and a V-0 UL-94 rate in PER. The composites containing only RDP showed a slight decrease, as seen in Table 1, while the mixed samples showed a small increase in LOI compared to the matrices. Except for the PER 4% P APP composite, all samples burned to the clamping during the vertical UL-94 test, most probably due to the high thermal conductivity of the included carbon fibres, which lead to HB UL-94 rates.

#### 3.5.3. MLC Results of the Reference and Flame Retarded Epoxy Resin Composites Containing 4% P

**Figure 4:**
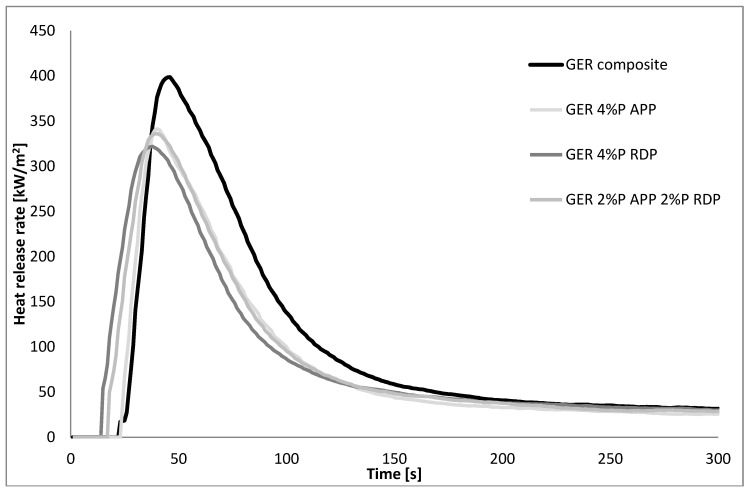
The heat release rate of the reference and flame retarded GER composites.

**Figure 5:**
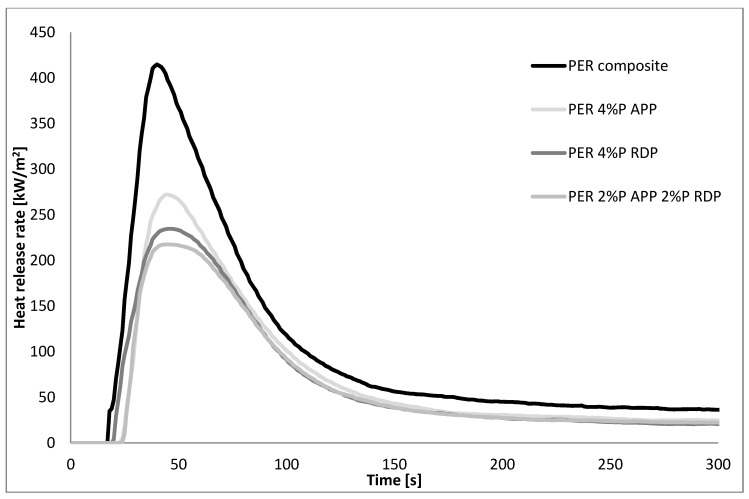
The heat release rate of the reference and flame retarded PER composites.

The MLC results of the composites seen in Figure 4 and Figure 5, Table 7 showed that the inclusion of 60 mass% of carbon fibre itself reduced the heat release rate to approximately 400 kW/m^2^ both in PER and GER and all heat release rate curves became single-peaked. The time to ignition tendencies remained similar to those of the matrices: the flame retarded GER composites ignited earlier than the reference composite, while in PER composites the time to ignition increased due to the addition of FRs. Also, similarly to the matrices, the fire performance of the same FRs was better in PER than in GER, although the reference composite had similar heat release rate characteristics. As for GER composites, GER 4% P RDP ignited at first, followed by the mixed FR composite and GER 4% P APP. It can be assumed that RDP started to act as FR in the early stages of degradation and this action lead to the lowest pHRR of 322 kW/m^2^. Furthermore, the solid-phase action of APP was hindered by the reinforcing carbon fibres, but the differences between the flame retarded samples are minor. Among the flame retarded PER composites, PER 4% P RDP ignited earliest (it is assumed again that the gas phase action starts in the early phase of degradation), leading to a reduced pHRR in comparison to the PER 4% P APP composite. In PER composites, the combination of APP and RDP had a synergistic effect, leading to a further decrease in pHRR to 218 kW/m^2^ and in THR to 18.2 MJ/m^2^. As for the amount of residues, it increased in all flame retarded composites compared to the reference composites, but no clear tendencies could be identified.

### 3.6. Dynamic Mechanical Properties of the Composites

In order to determine the impact of the additive flame retardants on the glass transition temperature and storage modulus of the composites we carried out dynamic mechanical analysis (DMA) as seen in Table 8.

The DMA results show that the plasticizing effect of liquid RDP is more distinct, when 4% P was added, the *T*_g_ decreased by 19 °C in GER and by 28 °C in PER composites. In the case of solid APP, in GER the decrease is only 1 °C, while in PER the *T*_g_ even increased by 11 °C. In the combined FR samples, APP compensated for the softening effect of RDP, similarly to the matrix results. As for the storage modulus, the values were compared below the *T*_g_, at room temperature, and in/above the region of *T*_g_. The addition of RDP caused the largest decrease in storage modulus in both systems and at both temperatures. We successfully compensated for this effect by combining RDP with APP: at 25 °C this combination even turned out to be synergistic concerning storage modulus, while at 75 °C the storage modulus of the mixed samples was between the samples containing only one additive.

## 4. Conclusions

We investigated the fire retardancy of a low viscosity glycerol- (GER) and a pentaerythritol (PER)-based EP and their carbon fibre reinforced composites applying ammonium polyphosphate (APP), acting in the solid phase, resorcinol bis(diphenyl phosphate) (RDP), acting predominantly in the gas phase, and their combination.

For preliminary fire performance screening, we prepared samples of gradually increasing phosphorus content (1%, 2%, 3%, 4%, and 5%) and mixed formulations with 2% P content from APP and 2% P content from RDP. Our results showed that at least 4% P content is necessary for a reasonable improvement in FR performance; the best overall results at this P content were achieved with GER 2% P APP 2% P RDP (LOI 28 *V*/*V*%, V-0), PER 4% P RDP (LOI 32 *V*/*V*%, V-0) and PER 2% P APP 2% P RDP (LOI 31 *V*/*V*%, V-0). Although the GER-based and the PER-based reference EP matrices have a similar initial LOI and horizontal burning rate, it is evident that with the same FR-content, PER delivers better fire performance.

We analyzed the compositions with 4% P content in detail. Parallel plate rheology investigations proved that all flame retarded systems are injectable at a temperature as low as 40 °C, except for the PER 4% P APP composition, which needs to be heated above 60 °C; this means that composites can be easily prepared by RTM and similar injection techniques. The DSC results of the EP matrices show that the plasticizing effect is more pronounced in the case of liquid RDP, and in the combined FR samples, APP slightly compensated for the softening effect of RDP. The reaction enthalpies related to the mass of the epoxy resin matrix indicated that the effects of APP and RDP are similar, and in mixed FR samples, the initiated decrease was slightly smaller than in the systems containing only one FR. The TGA results showed that RDP, acting mainly in the gas phase at the beginning of degradation, shifts the beginning of thermal degradation to lower temperatures. The initial maximum mass loss rate of GER is much higher than that of PER, which is consistent with the LOI results of these matrices. The FRs increase the amount of solid residues at 800 °C in the following order: 4% P APP < 4% P RDP < 2% P APP 2% P RDP, suggesting that the combination of these two FRs is advantageous in terms of thermal stability as well. The mass loss calorimetry results indicate that the trifunctional GER has approx. 1.5 times higher pHRR than the tetrafunctional PER, which is in good agreement with the LOI results. In GER a clear synergistic effect was observed, in terms of pHRR and TTI when APP and RDP were applied together, while in PER the 4% P APP sample had the lowest pHRR followed closely by the mixed sample. Both in PER and GER the mixed FR systems had the lowest THR value.

As for the composites, the incorporation of carbon fibres itself increased the LOI of GER and PER, but except for the PER 4% P APP composite, all samples burned to the clamping during the vertical UL-94 test, most probably due to the high thermal conductivity of the included carbon fibres. Similarly, the inclusion of 60 mass% of carbon fibres itself reduced the pHRR to approximately 400 kW/m^2^ in both systems, and the fire performance of the same FRs was better in PER than in GER. In both matrices, the 4% P RDP composite ignited at first, as RDP started to act as a FR in the early stages of degradation, while the solid-phase action of APP was hindered by the reinforcing carbon fibres. In PER composites, the combination of APP and RDP had a synergistic effect, leading to further decrease in pHRR to 218 kW/m^2^ and in THR to 18.2 MJ/m^2^. The DMA results indicated that the combination of APP and RDP compensated for the effect that RDP decreased the *T*_g_ and proved to be synergistic in the terms of storage modulus values at room temperature.

## Figures and Tables

**Table 1:** Limiting oxygen index (LOI) and UL-94 results of the reference and flame retarded trifunctional glycerol (GER) and tetrafunctional pentaerythritol-based (PER) matrices. APP: ammonium polyphosphate; RDP: resorcinol bis(diphenyl phosphate).

Matrix	LOI (*V*/*V*%)	UL-94 (Burning rate)	Matrix	LOI (*V*/*V*%)	UL-94 (Burning rate)
GER	22	HB (27 mm/min)	PER	23	HB (32 mm/min)
GER 1% P APP	23	HB	PER 1% P APP	27	HB
GER 2% P APP	23	HB	PER 2% P APP	32	HB
GER 3% P APP	25	HB	PER 3% P APP	32	HB
GER 4% P APP	27	HB	PER 4% P APP	32	V-1
GER 5% P APP	28	HB	PER 5% P APP	32	V-0
GER 1% P RDP	24	HB (23 mm/min)	PER 1% P RDP	25	HB (15 mm/min)
GER 2% P RDP	26	HB	PER 2% P RDP	26	HB
GER 3% P RDP	26	HB	PER 3% P RDP	29	HB
GER 4% P RDP	29	V-1	PER 4% P RDP	32	V-0
GER 5% P RDP	30	V-0	PER 5% P RDP	32	V-0
GER 2% P APP 2% P RDP	28	V-0	PER 2% P APP 2% P RDP	31	V-0

Average standard deviation of the measured burning rate: ±1 mm/min.

**Table 2:** Effect of the additive flame retardants on the viscosity of GER and PER matrix samples.

	Viscosity * (mPa·s)			
**Matrix**	**25 °C**	**40 °C**	**60 °C**	**80 °C**
GER	**171**	62	50	79
GER 4% P APP	**269**	**120**	84	93
GER 4% P RDP	613	**229**	**149**	**112**
GER 2% P APP 2% P RDP	**252**	**160**	**205**	**124**
PER	603	**293**	**116**	57
PER 4% P APP	1078	564	313	**149**
PER 4% P RDP	506	**236**	13	5
PER 2% P APP 2% P RDP	663	**211**	**109**	5

* Viscosities suitable for injection are displayed with bold numbers.

**Table 3:** Effect of the additive flame retardants on the glass transition temperature, reaction enthalpy and temperature of the exothermic peak in GER and PER matrix samples.

Matrix	Glass transition temperature (°C)	Reaction enthalpy		Temperature of exothermic peak (°C)
		(J/g)	(J/g epoxy)	
GER	98	410	410	88
GER 4% P APP	82	280	320	100
GER 4% P RDP	45	214	340	85
GER 2% P APP 2% P RDP	59	269	358	86
PER	114	378	378	93
PER 4% P APP	114	303	346	89
PER 4% P RDP	81	192	305	89
PER 2% P APP 2% P RDP	83	264	351	85

**Table 4:** Thermogravimetric analysis (TGA) results of the reference and flame retarded GER and PER matrix samples.

Matrix	*T*_−5%_ (°C)	*T*_−50%_ (°C)	d*T*G_max_ (%/°C)	*T*_dTGmax_ (°C)	Char yield at 800 °C (%)
GER	304	330	7.2	305	2.1
GER 4% P APP	297	334	4.2	300	12.7
GER 4% P RDP	241	317	1.1	285	14.1
GER 2% P APP 2% P RDP	269	328	1.0	284	20.3
PER	293	334	2.8	294	1.6
PER 4% P APP	288	342	1.0	314	12.5
PER 4% P RDP	273	328	1.0	296	13.8
PER 2% P APP 2% P RDP	280	333	1.0	292	15.0

*T*_−5%_: temperature at 5% mass loss, *T*_−50%_: temperature at 50% mass loss; d*T*G_max_: maximum mass loss rate; *T*_dTGmax_: the temperature belonging to the maximum mass loss rate.

**Table 5:** Mass loss calorimetry (MLC) results of the reference and flame retarded PER and GER matrices.

Matrix	TTI (s)	pHRR (kW/m^2^)	Time of pHRR (s)	THR (MJ/m^2^)	Residue (Mass%)
GER	26	1101	83	90.6	0
GER 4% P APP	29	627	58	63.1	12
GER 4% P RDP	25	600	108	75.7	14
GER 2% P APP 2% P RDP	28	408	116	60.5	10
PER	13	706	67	103.5	0
PER 4% P APP	23	358	123	77.4	18
PER 4% P RDP	19	346	75	69.5	10
PER 2% P APP 2% P RDP	20	445	96	60.5	4

TTI: time to ignition, pHRR: peak of heat release rate, THR: total heat release. Average standard deviation of the measured mass loss calorimeter values: TTI: ±3, pHRR: ±30, time of pHRR: ±5, residue: ±2.

**Table 6:** LOI and UL-94 results of the reference and flame retarded GER and PER composites.

Composite	LOI (*V*/*V*%)	UL-94	Composite	LOI (*V*/*V*%)	UL-94
GER	25	HB	PER	31	HB
GER 4% P APP	31	HB	PER 4% P APP	37	V-0
GER 4% P RDP	27	HB	PER 4% P RDP	29	HB
GER 2% P APP 2% P RDP	29	HB	PER 2% P APP 2% P RDP	32	HB

**Table 7:** Mass loss calorimetry of the reference and flame retarded PER and GER composites.

Composite	TTI (s)	pHRR (kW/m^2^)	Time of pHRR (s)	THR (MJ/m^2^)	Residue (Mass%)
GER	23	399	46	28.6	24.8
GER 4% P APP	24	341	40	23.0	32.6
GER 4% P RDP	15	322	38	24.3	33.0
GER 2% P APP 2% P RDP	18	336	40	24.5	34.2
PER	18	415	40	29.2	25.8
PER 4% P APP	24	272	45	20.5	32.6
PER 4% P RDP	20	235	46	19.3	34.8
PER 2% P APP 2% P RDP	23	218	45	18.2	31.0

TTI: time to ignition, pHRR peak of heat release rate, THR: total heat release. Average standard deviation of the measured mass loss calorimeter values: TTI: ±3, pHRR: ±30, time of pHRR: ±5, residue: ±2.

**Table 8:** The glass transition temperature and storage modulus of the reference and flame retarded composites.

Composite	Glass transition temperature (°C)	Storage modulus at 25 °C (MPa)	Storage modulus at 75 °C (MPa)
GER	55	71731	17187
GER 4% P APP	54	80141	26076
GER 4% P RDP	36	39997	10906
GER 2% P APP 2% P RDP	49	85598	23631
PER	69	85857	48002
PER 4% P APP	80	60935	39455
PER 4% P RDP	41	36146	7331
PER 2% P APP 2% P RDP	63	61416	21372
